# The influences of anesthesia methods on some complications after orthopedic surgery: a Bayesian network meta-analysis

**DOI:** 10.1186/s12871-019-0701-2

**Published:** 2019-04-09

**Authors:** Yuqing Zeng, Junming Wan, Haiyong Ren, Jianwei Lu, Fuhua Zhong, Shu Deng

**Affiliations:** 1 The First Clinical Medical College, Guangzhou University of Chinese Medicine, 16 Jichang Road, Baiyun District, Guangzhou, 510405 Guangdong Province People’s Republic of China; 2 Department of Orthopaedics, Tongde Hospital of Zhejiang Province, Hangzhou, Zhejiang Province People’s Republic of China; 3 Department of Hematology, The First Affliated Hospital of Zhejiang Chinese Medical University, 54 Youdian Road, Hangzhou, Zhejiang Province People’s Republic of China

**Keywords:** Orthopedic surgery, Nerve block analgesia, Local infiltration analgesia, Interscalene block, Complication

## Abstract

**Background:**

Although several anesthesia procedures have been explored for orthopedic surgery, the complications of anesthesia remain not well resolved. This study aimed to explore the influence of different anesthesia methods on the complications after orthopedic surgery.

**Methods:**

According to the searching strategy, anesthesia associated studies in orthopedic surgery were screened from Pubmed, Embase, and the Cochrane Library up to Mar. 10th, 2018. Then, complications and demographic data were extracted and quality of studies was assessed using Cochrane Collaboration recommendations. ADDIS software was used to perform the network meta-analysis. Pooled effect size was calculated using random effective model or consistency model, and presented with odds ratio (OR) and 95% confidence interval (CI).

**Results:**

According to the selective criteria, a total of 23 studies with 2393 patients were enrolled in this study. Quality assessment revealed all studies had an ordinary quality. Network meta-analyses revealed that nerve block analgesia (NBA) presented a lower effect on the occurrence of post-operative nausea or vomiting (PONV; OR = 0.17, 95% CI: 0.06–0.39) and urine retention (OR = 0.07, 95% CI: 0.01–0.37) compared with epidural anesthesia (EA). Interscalene block (ISB) and local infiltration analgesia (LIA) could significantly reduce the occurrence of back pain compared with EA (OR = 0.00, 95% CI = 0.00–0.30; OR = 0.00, 95% CI = 0.00–0.25).

**Conclusion:**

NBA presented an effective role in reliving the occurrence of PONV and urine retention, and ISB and LIA relieved the back pain compared with EA after orthopedic surgery.

## Background

Since it emerged in the eighteenth century, the discipline of orthopedic surgery has been remarkably developed [1]. Till now, several orthopedic surgeries have been explored, including total knee replacement, hip fracture, and total hip replacement [2]. However, there are still some deficiencies to limit the application of orthopedic surgery in clinic, such as pain control, prevention of post-operative nausea or vomiting (PONV), rapid recovery, cognitive impairment, and surgical site infection [3–5]. Specifically, anesthesia is a common procedure during orthopedic surgery, which could affect the temperature regulation, infection, bleeding, oxygen consumption, and other complications to influence the outcome of orthopedic surgery [6]. Therefore, it is important to innovate appropriate anesthesia manner to improve the outcomes and prognosis of orthopedic surgery.

With the development of few decades, although several anesthesia manners have been explored for orthopedic surgery, the complications of anesthesia are still not well resolved. A previous study has revealed that patients managed with general anesthesia perform a low risk of complications compared with patients undergoing spinal anesthesia during the total knee arthroplasty [7]. However, compared with the general anesthesia, regional anesthesia presents a better outcome than general anesthesia in total hip arthroplasty, including reductions of deep surgical site infection, length of hospital stay, and pulmonary complication [8]. Moreover, Stundner et al. have revealed that neuraxial anesthesia reduces the occurrence rates of blood transfusions and morbidity in the perioperative period of compared with general anesthesia for simultaneous bilateral total knee arthroplasty [9]. In addition, Ewan et al. have documented that general anesthesia increases the risk of post-operative cognitive dysfunction compared with other anesthesia methods [10]. Considering of these evidences, there is still no clear consensus in anesthesia during orthopedic surgery.

In the current study, a network meta-analysis was performed to comprehensively estimate the effects of different anesthesia manners, such as general anesthesia on the outcomes of orthopedic surgery. According to this analysis, we hope to provide some new insights for improving the outcomes of orthopedic surgery.

## Methods

### Data sourcing

According to the searching strategy, studies focused on the associations between anesthesia methods and adverse effects after orthopedic surgery published in English were downloaded from the Pubmed (http://www.ncbi.nlm.nih.gov/pubmed), Embase (http://www.embase.com), and the Cochrane Library (http://www.cochranelibrary.com) databases. The searching date was ranged from its recording to Mar. 10th, 2018. The searching strategy was designed as follows: “general anesthesia” (OR “general anaesthesia” OR “local anesthesia” OR “topical anesthesia” OR “local anaesthesia” OR “toponarcosis” OR “medullary anesthesia” OR “rachianalgesia” OR “rachianesthesia” OR “medullary narcosis” OR “spinal anesthesia” OR “rhachiaesthesia” OR “rhachianalgesia” OR “lumbar anesthesia” OR “epidural anesthesia” OR “epidural block” OR “epidural anaesthesia” OR “caudal anaesthesia” OR “caudal anesthesia” OR “caudalanaesthesia” OR “infiltration analgesia” OR “intrathecal analgesia”) AND “orthopedics” (OR “orthopedic” OR “osteology”) AND “Rando”.

### Inclusive and exclusive criteria

In the present study, studies were included if they met the following terms: (1) published in English; (2) reported on the influences of different anesthesia methods on the effective of patients (P) undergoing orthopedic surgery; (3) patients in different groups receiving different anesthesia methods (Intervention, I; and Control, C); (4) study outcome variables including PONV, urine retention, back pain, sore throat, and headache, and so on (Outcomes, O); and (5) randomized controlled trial (RCT; S). Studies were excluded if they were met the following criteria: (1) incomplete data which could not be used for statistical analysis; (2) reviews, letters and comments; (3) for duplicate publication or data used for several studies, only the study with complete data was included, and others were excluded.

### Data extraction and quality assessment

Data was independently extracted from the included studies by two censors in this study, respectively. The extracted information included the first author, published year, study year, study area, anesthesia method, sample size in different groups, length of operation, and the demographic characteristics of included patients, including age, gender, height, weight and so on. Quality of the enrolled studies were assessed using the Cochrane Collaboration recommendations recommended by the Cochrane system [11]. During the data extraction and quality assessment, divergences were solved by discussing with the third censors.

### Statistical analyses

ADDIS is a non-programming software based on Bayesian framework, and can be used for data evaluation using the Markov chain Monte Carlo theory [12, 13]. All data in the current study was analyzed using the ADDIS software (version 1.16.5), and presented with odd ratio (OR) and 95% confidence interval (CI). For *P* < 0.05 in node-splitting analysis, the random effects model was used to calculate pooled effect size; otherwise, the consistency model were used to calculate the pooled effect size. Convergence degree of model was estimated using Brooks-Gelman-Rubin method, and presented with the potential scale reduction factor (PSRF). The more PSRF approximate to 1, the better convergence was obtained [14].

## Results

### Characteristics of enrolled studies

**Fig. 1:**
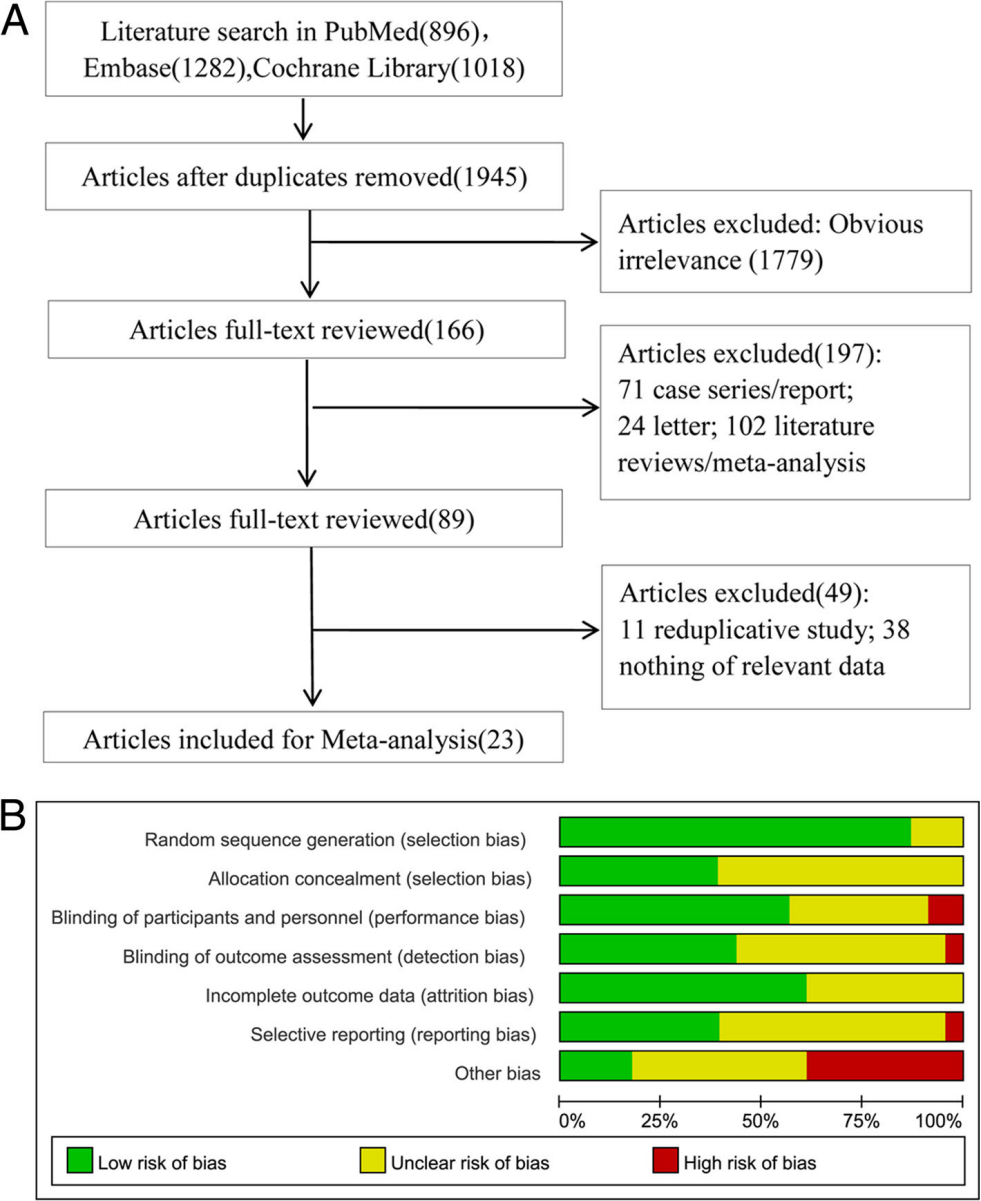
Study enrollment and quality assessment. **a**, Flow chart of study enrollment; **b**, Quality assessment of enrolled studies

According to the searching strategy, a total of 3196 studies were recruited in this study. After removing the repetitions, 1945 studies were obtained. Following this, 1779 papers among 1945 were rejected after scanning title and abstract. Subsequently, 143 studies among remains were removed after reviewing the full text. Finally, 23 studies were obtained [15–37] and the process of study enrollment was presented in Fig. 1A.

Characteristics of the enrolled studies were summarized in Table 1. For these enrolled studies, the published years of them were ranged from 1978 to 2017, and the research areas were concentrated on Germany, Japan, America, China, French, and Turkey. A total of 2393 patients were enrolled in this study, including 753 in general anesthesia (GA) group, 215 in epidural anesthesia (EA) group, 473 in local infiltration analgesia (LIA) group, 238 in nerve block analgesia (NBA) group, 630 in spinal anesthesia (SA) group, 65 in interscalene block (ISB) group, and 81 in GA + ISB group. Moreover, the number of male patients was slightly higher than female patients, but there was no significantly difference for gender ratio in each study. In addition, there were no significantly difference identified in the age, height, weight, and length of operation among groups. Quality assessment indicated that the enrolled studies presented an ordinary quality (Fig. 1B). Despite the random sequence generation (selection bias), most of studies didn’t report more information on other quality assessment terms.

**Table 1:** Characteristics of enrolled studies

Author	Public Year	Location	Study Yeat	Group	N	Age (years)*	Male/Female	Weight(kg)*	Height (cm)*	Length of operation (min)*
Arcioni R	2007	Italy	2004.9–2004.12	EA	25	59.1 ± 19.4	12/13	68.0 ± 8.7	166.2 ± 8.4	NA
				SA	23	60.2 ± 21.2	9/14	68.3 ± 10.4	164.7 ± 7.9	NA
Kuchalik J	2013	Sweden	NA	SA	39	66(51–84)	23/16	84 ± 23	170 ± 10	106 ± 17
				LIA	39	67(50–85)	21/18	86 ± 20	173 ± 8	112 ± 28
Dadure C	2006	France	2001.7–2002.12	EA	27	1–12	NA	7–56	72–160	65–190
				NBA	25	1–11	NA	10–52	80–151	45–180
Dunn WR	2006	USA	NA	LIA	18	Mean:51	11/7	Mean:75.0	Mean:170.3	Mean:27.4
				SA	14	Mean:55	5/9	Mean:74.2	Mean:169.6	Mean:30.9
Hadzic A	2005	USA	2000.4–2002.3	ISB	25	49 ± 13	17/8	85 ± 20	173 ± 10	127 ± 35
				GA	25	49 ± 12	13/12	86 ± 21	172 ± 10	147 ± 49
Hadzic A	2004	USA	NA	NBA	25	45 ± 15	12/13	81 ± 18	173 ± 10	72(50–165)
				GA	25	40 ± 16	11/14	77 ± 15	170 ± 10	70(30–330)
Janssen H	2014	Germany	NA	GA	42	51 ± 10	19/23	80 ± 14	170 ± 7	56.0 ± 12.4
				GA + ISB	41	53 ± 9	18/23	81 ± 16	170 ± 8	46.0 ± 15.3
Karaarslan S	2015	Turkey	NA	SA	30	43 ± 13	13/17	75 ± 13	170 ± 7	79 ± 22
				NBA	30	43 ± 10	19/11	77 ± 16	169 ± 9	85 ± 23
Krobbuaban B	2005	Thailand	NA	SA	86	41 ± 20	45/41	58 ± 13	161 ± 8	86 ± 52
				GA	85	38 ± 17	47/38	56 ± 8	168 ± 7	71 ± 15
Lehmann LJ	2014	Germany	2011.7–2012.5	GA	40	54.1 ± 11.7	22/18	83.3 ± 16.6	172.6 ± 10.7	NA
				ISB	40	49.3 ± 13.6	27/13	88.2 ± 19.2	172.2 ± 9.9	NA
				GA + ISB	40	53.8 ± 15.2	18/22	81.5 ± 16.3	169 ± 9.8	NA
Nagafuchi M	2015	Japan	2012.10–2013.7	NBA	17	72 ± 10	2/15	55 ± 8.2	NA	71 ± 15
				LIA	16	73 ± 5.9	3/13	62 ± 12.5	NA	81 ± 20
Seeberger MD	1994	Switzerland	NA	SA	96	33.7 ± 12.3	73/23	73.7 ± 12.6	174.2 ± 5.3	62 ± 35
				EA	96	32.0 ± 9.0	67/29	72.7 ± 11.0	174.7 ± 5.4	68 ± 46
Spangehl MJ	2015	NA	NA	NBA	79	67.8 ± 7.9	41/37	NA	NA	NA
				LIA	81	67.7 ± 7.2	48/43	NA	NA	NA
Standl T	1996	Germany	NA	SA	221	41.3 ± 17.8	112/109	70.4 ± 11	170.5 ± 8	120 ± 19
				GA	212	43.2 ± 17.3	106/106	70.9 ± 9	172.1 ± 6	116 ± 5
Gi E	2014	Japan	NA	LIA	25	77 ± 7	24/1	61 ± 13	149 ± 7	174 ± 23
				NBA	24	78 ± 5	21/3	64 ± 13	151 ± 7	173 ± 27
Bigler D	1985	NA	NA	GA	20	77.6 ± 2.3	5/15	NA	NA	59 ± 10
				SA	20	80.1 ± 1.6	2/18	NA	NA	67 ± 8
Hole A	1980	Norway	NA	GA	31	71.7(61–82)	11/20	NA	NA	207 ± 6
				EA	29	69.9(56–84)	10/19	NA	NA	190 ± 6
Kudoh A	2004	Japan	NA	SA	75	75.9 ± 4.0	69/6	60.4 ± 8.7	151.3 ± 7.3	106.7 ± 31.5
				GA	75	75.1 ± 4.2	66/9	59.2 ± 5.9	149.3 ± 5.4	104.2 ± 11.8
McLaren AD	1978	UK	NA	GA	29	76 ± 9.7	NA	NA	NA	NA
				SA	26	75.6 ± 10.3	NA	NA	NA	NA
Tanikawa H	2014	Japan	NA	LIA	23	71(69–76)	19/4	55.0(53.5–66.0)	151(148–152)	82.4 ± 26.0
				NBA	23	72(67.5–76.5)	20/3	54.5(48.0–66.5)	150(143.5–155.5)	75.0 ± 27.3
Trker G	2003	Turkey	NA	EA	15	62.2 ± 6.6	9/6	72.2 ± 7.5	166.6 ± 3	129.2 ± 26.4
				NBA	15	62.3 ± 7.2	8/7	73.7 ± 6.3	167.4 ± 4.4	131.3 ± 18.7
Wang H	2017	China	2008.1–2015.12	GA	169	52.9 ± 9.7	89/80	NA	NA	52.5 ± 9.3
				LIA	187	51.4 ± 9.1	93/94	NA	NA	48.1 ± 9.9
Yukawa Y	2005	Japan	NA	LIA	22	58.9 ± 14.5	15/7	60.3 ± 9.5	159.2 ± 7.9	160.7 ± 27.0
				EA	23	59.1 ± 15.2	10/13	59.0 ± 9.7	160.1 ± 8.7	157.5 ± 29.5

Abbreviations: PONV: post-operative nausea or vomiting; GA: general anesthesia; LIA: local infiltration analgesia; ISB: interscalene block; EA: epidural anesthesia; NBA: nerve block analgesia; SA: spinal anesthesia; min: minutes; *: mean ± standard deviation/median(min-max)

### Network meta-analyses for adverse effects after orthopedic surgery

According to the extracted data, parameters of ADDIS were set as follows: Number of chains: 4, Tuning iterations: 20000, Simulation iterations: 50000, Thinning interval: 10, Inference samples: 10000, Variance scaling factor: 2.5, and the network meta-analyses for PONV, urine retention, sore throat, back pain and headache were analyzed.

### Network analysis for PONV

**Fig. 2:**
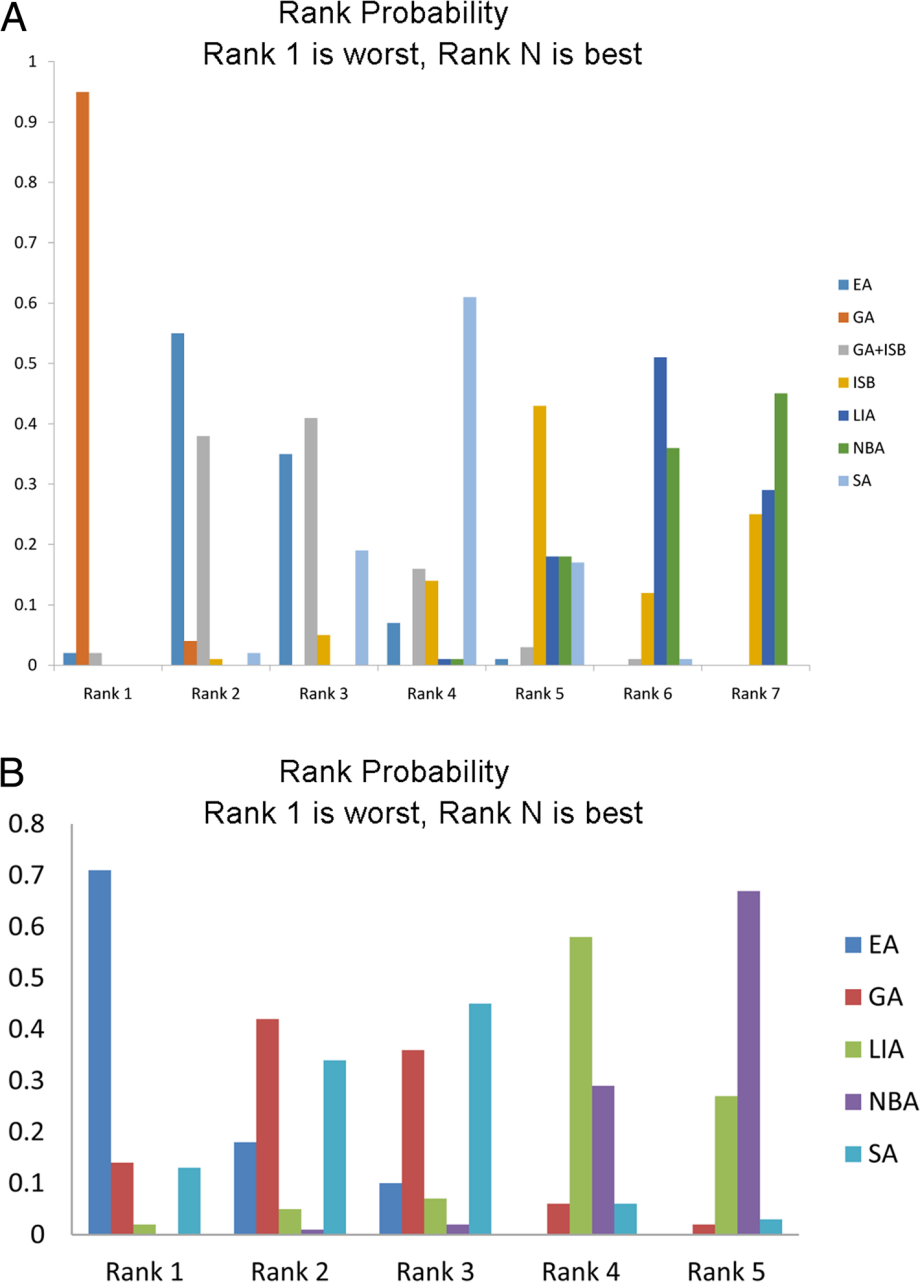
Network meta-analyses for PONV and urine retention. **a**, Network meta-analyses for PONV; **b**, Network meta-analyses for urine retention. PONV: post-operative nausea or vomiting; GA: general anesthesia; LIA: local infiltration analgesia; ISB: interscalene block; EA: epidural anesthesia; NBA: nerve block analgesia; SA: spinal anesthesia

For PONV, the PSRF value was ranged from 1.00 to 1.01, indicating model had a good convergence. The node-splitting analysis presented that *P* values of all comparisons were more than 0.05 (Table 2A), and the consistency model was used to calculate the pooled effect sizes. The result presented that NBA had lowest influence on PONV after orthopedic surgery, and GA presented the worst effect on PONV after orthopedic surgery (Fig. 2A). Compared with NBA group, SA (OR = 0.31, 95%CI: 0.10, 0.86), EA (OR = 0.17, 95%CI: 0.06–0.39), GA (OR = 0.07, 95%CI: 0.02–0.18), and GA + IBS (OR = 0.19, 95%CI: 0.04–0.81) presented significantly worse effect on PONV after orthopedic surgery (Table 3A).

**Table 2:** Node-splitting analysis for PONV and urine retention

Name	Direct Effect	Indirect Effect	Overall	*P*-Value
A: PONV				
EA, GA	1.02 (−0.47, 2.42)	0.88 (−0.31, 2.09)	0.91 (0.02, 1.88)	0.89
EA, SA	0.25 (−1.15, 1.85)	−1.24 (−2.46, −0.20)	−0.68 (−1.58, 0.25)	0.10
EA, LIA	−2.46 (−4.33, − 0.74)	−1.38 (−2.44, − 0.38)	−1.74 (−2.67, − 0.89)	0.29
EA, NBA	−2.03 (−4.03, − 0.76)	−1.46 (− 2.79, − 0.21)	−1.80 (− 2.82, − 0.93)	0.47
GA, LIA	− 2.43 (− 4.62, − 0.74)	−2.77 (−3.90, − 1.76)	−2.64 (−3.70, − 1.75)	0.78
GA, NBA	−1.80 (− 4.09, − 0.11)	− 2.95 (− 4.22, − 1.99)	− 2.71 (−3.88, − 1.74)	0.41
GA, SA	−1.78 (− 2.62, − 0.96)	−0.91 (− 2.41, 0.54)	−1.57 (− 2.27, − 0.88)	0.30
GA + ISB, ISB	− 1.03 (− 3.23, 0.72)	−1.62 (− 3.68, 0.32)	−1.23 (− 2.76, 0.23)	0.65
LIA, SA	0.83 (− 0.50, 2.13)	1.29 (0.13, 2.66)	1.08 (0.20, 2.04)	0.62
LIA, NBA	− 0.08 (− 0.90, 0.65)	0.04 (−1.68, 1.49)	−0.07 (− 0.81, 0.63)	0.89
B: Urine retention				
EA, GA	−0.47 (− 2.91, 1.86)	−1.37 (− 4.68, 1.53)	−0.68 (− 2.52, 0.87)	0.60
EA, NBA	−2.93 (−5.49, − 0.99)	− 1.35 (−5.92, 2.38)	−2.59 (− 4.56, − 1.00)	0.45
EA, SA	−0.66 (− 4.75, 2.23)	−0.67 (− 3.34, 1.44)	−0.76 (− 2.71, 0.86)	0.96
GA, SA	0.20 (− 1.68, 2.06)	− 0.94 (− 4.47, 2.55)	−0.08 (− 1.60, 1.43)	0.55
NBA, SA	0.81 (− 2.34, 4.35)	2.50 (− 0.56, 5.47)	1.84 (− 0.26, 3.93)	0.48

Abbreviations: PONV: post-operative nausea or vomiting; GA: general anesthesia; LIA: local infiltration analgesia; ISB: interscalene block; EA: epidural anesthesia; NBA: nerve block analgesia; SA: spinal anesthesia. Data was presented with odds ratio and 95% confidence interval

**Table 3:** Network meta-analyses for PONV and urine retention

A: PONV						
EA	2.48 (1.02, 6.55)	0.85 (0.23, 3.54)	0.26 (0.05, 1.17)	0.18 (0.07, 0.41)	0.17 (0.06, 0.39)	0.51 (0.21, 1.29)
0.40 (0.15, 0.98)	GA	0.34 (0.13, 0.97)	0.10 (0.03, 0.36)	0.07 (0.02, 0.17)	0.07 (0.02, 0.18)	0.21 (0.10, 0.42)
1.17 (0.28, 4.42)	2.91 (1.03, 7.79)	GA + ISB	0.29 (0.06, 1.25)	0.20 (0.05, 0.76)	0.19 (0.04, 0.81)	0.59 (0.17, 1.94)
3.81 (0.86, 18.19)	9.67 (2.78, 36.55)	3.42 (0.80, 15.77)	ISB	0.68 (0.14, 3.40)	0.63 (0.11, 3.34)	2.02 (0.48, 8.87)
5.71 (2.44, 14.43)	13.98 (5.74, 40.29)	4.91 (1.32, 21.15)	1.48 (0.29, 7.31)	LIA	0.93 (0.44, 1.87)	2.95 (1.22, 7.69)
6.02 (2.53, 16.79)	15.08 (5.69, 48.64)	5.24 (1.24, 25.69)	1.58 (0.30, 8.71)	1.07 (0.53, 2.26)	NBA	3.22 (1.16, 9.67)
1.97 (0.78, 4.85)	4.82 (2.40, 9.66)	1.69 (0.52, 5.87)	0.49 (0.11, 2.08)	0.34 (0.13, 0.82)	0.31 (0.10, 0.86)	SA
B: Urine retention						
EA	0.51 (0.08, 2.38)	0.10 (0.01, 1.18)		0.07 (0.01, 0.37)	0.47 (0.07, 2.37)	
1.98 (0.42, 12.43)	GA	0.21 (0.01, 3.65)		0.15 (0.02, 1.36)	0.92 (0.20, 4.18)	
9.84 (0.84, 151.97)	4.87 (0.27, 82.03)	LIA		0.71 (0.11, 5.12)	4.34 (0.25, 77.47)	
13.36 (2.73, 95.12)	6.83 (0.74, 57.95)	1.41 (0.20, 9.26)		NBA	6.27 (0.77, 51.01)	
2.14 (0.42, 15.04)	1.09 (0.24, 4.94)	0.23 (0.01, 3.95)		0.16 (0.02, 1.29)	SA	

PONV: post-operative nausea or vomiting; GA: general anesthesia; LIA: local infiltration analgesia; ISB: interscalene block; EA: epidural anesthesia; NBA: nerve block analgesia; SA: spinal anesthesia. Data was presented with odds ratio and 95% confidence interval

### Network analysis for urine retention

The PSRF for urine retention was ranged from 1.00 to 1.02 indicating a good convergence for PSRF. Node-splitting analysis presented that *P* > 0.05, thus, the consistency model was used to calculate the pooled effect size of urine retention (Table 2B). The network analysis presented that the NBA group presented the lowest incidence of urine retention, and its incidence was significantly lower than that in the EA group (OR = 0.07, 95%CI: 0.01–0.37, Table 3B and Fig. 2B).

### Analysis for sore throat

**Fig. 3:**
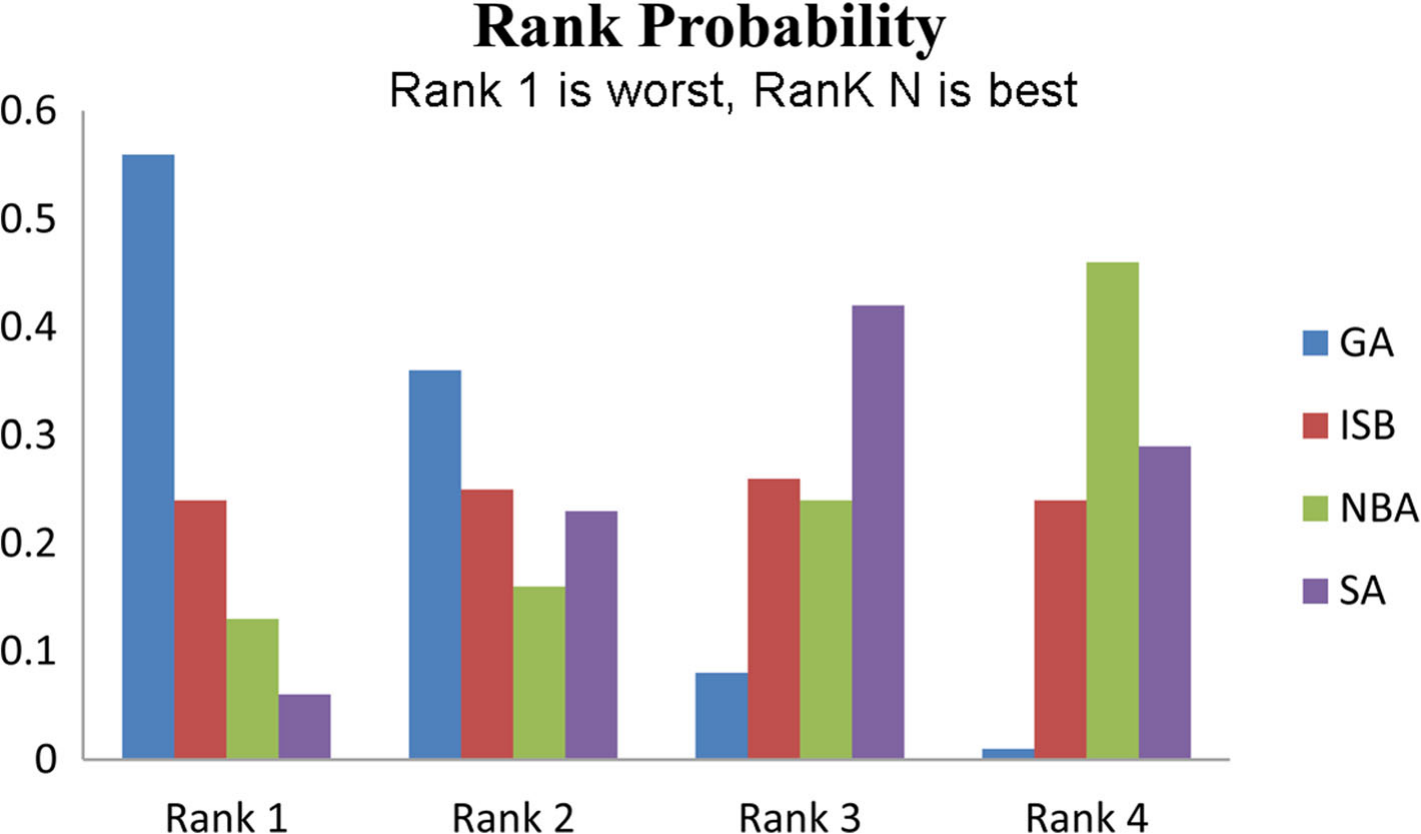
Network meta-analyses for sore throat. GA: general anesthesia; LIA: local infiltration analgesia; ISB: interscalene block; NBA: nerve block analgesia; SA: spinal anesthesia

For sore throat, all PSRF values were 1.01, indicating a good convergence. Because no closed ring formed, consistency model was utilized to calculate the pooled side effect of sore throat. The analytical results presented that both the SA and NBA groups had lower incidences of sore throat, but no significant differences were identified compared with other groups (Fig. 3**,** Table 4).

**Table 4:** Network meta-analysis for sore throat

GA	0.18 (0.00, 107.05)	0.05 (0.00, 36.33)	0.09 (0.00, 4.78)
5.43 (0.01, 3647.30)	ISB	0.25 (0.00, 2894.67)	0.48 (0.00, 868.90)
20.89 (0.03, 21,288.40)	3.93 (0.00, 56,736.22)	NBA	1.74 (0.00, 5921.00)
11.51 (0.21, 588.42)	2.09 (0.00, 4009.84)	0.57 (0.00, 1205.54)	SA

GA: general anesthesia; ISB: interscalene block; NBA: nerve block analgesia; SA: spinal anesthesia. Data was presented with odds ratio and 95% confidence interval

### Analysis for back pain

**Fig. 4:**
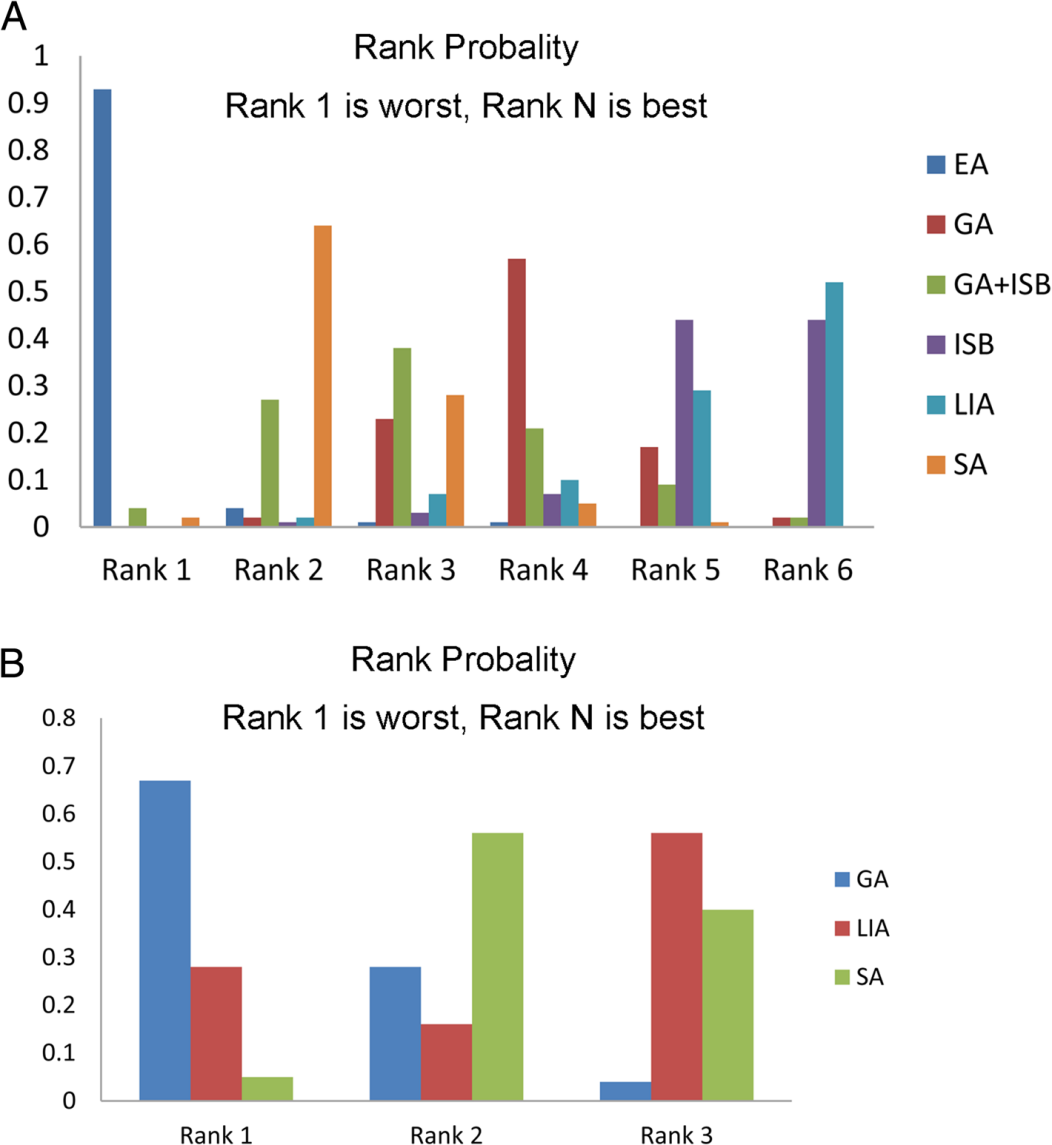
Network meta-analyses for back pain and headache. **a**, Network meta-analyses for back pain; **b**, Network meta-analyses for headache GA: general anesthesia; LIA: local infiltration analgesia; ISB: interscalene block; EA: epidural anesthesia; SA: spinal anesthesia.

For back pain, all PSRF values were 1.01, indicating a good convergence. Because no closed ring formed, consistency model was utilized to calculate the pooled side effect of back pain. Compared with the EA group, both ISB (OR = 0.00, 95%CI: 0.00–0.30) and LIA (OR = 0.00, 95%CI: 0.00–0.25) groups presented lower incidences of back pain; however, no other significant difference was identified in comparison between other groups (Fig. 4A**,** Table 5A).

**Table 5:** Network meta-analyses for back pain and headache

A: Back pain					
EA	0.02 (0.00, 0.57)	0.04 (0.00, 2.38)	0.00 (0.00, 0.30)	0.00 (0.00, 0.25)	0.06 (0.00, 1.38)
51.32(1.75,3339.72)	GA	1.79 (0.14, 23.31)	0.20 (0.01, 3.35)	0.18 (0.00, 5.98)	2.98 (0.75, 20.40)
27.51(0.42,4086.82)	0.56 (0.04, 7.20)	GA + ISB	0.12 (0.01, 1.81)	0.10 (0.00, 8.09)	1.69 (0.10, 43.87)
267.22(3.36,44,447.31)	4.89 (0.30, 104.69)	8.53 (0.55, 176.80)	ISB	0.82 (0.01, 96.19)	15.26 (0.73, 591.84)
306.91(3.94,62,665.12)	5.69 (0.17, 374.21)	10.33(0.12,1263.02)	1.22 (0.01, 173.76)	LIA	17.27 (0.82, 906.94)
15.74 (0.72, 650.35)	0.34 (0.05, 1.33)	0.59 (0.02, 9.74)	0.07 (0.00, 1.37)	0.06 (0.00, 1.22)	SA
B: Headache					
GA	0.32 (0.00, 25.48)		0.42 (0.10, 1.60)		
3.17 (0.04, 217.06)	LIA		1.39 (0.02, 63.62)		
2.40 (0.63, 10.17)	0.72 (0.02, 55.14)		SA		

GA: general anesthesia; LIA: local infiltration analgesia; ISB: interscalene block; EA: epidural anesthesia; SA: spinal anesthesia. Data was presented with odds ratio and 95% confidence interval

### Analysis for headache

For back pain, all PSRF values were 1.01, indicating a good convergence. Because no closed ring formed, consistency model was utilized to calculate the pooled side effect of back pain. The network analysis presented that LIA group had the lowest incidence of headache, but no significant difference was revealed compared with other groups (Fig. 4B**,** Table 5B).

## Discussion

According to the selective criteria, a total of 23 studies with 2393 patients were enrolled in this study. With the network meta-analysis, patients undergoing NBA presented lower occurrence rates of PONV and urine retention compared with patients managed with SA, EA, GA, and GA + ISB during the perioperative period of orthopedic surgery. Meanwhile, patients managed with ISB and LIA were presented a significant lower occurrence rate of back pain compared with patients undergoing EA. However, there was no significant difference identified in the occurrence of headache among these groups.

NBA is a common anesthesia method utilized in orthopedic surgery, such as total knee arthroplasty [38], shoulder arthroscopy [39], and hip fracture [40]. It has been revealed that nerve blocks may present some benefits in lower risk of PONV, enhanced pain relief and earlier discharge [41, 42]. Park et al. have demonstrated that interscalene brachial plexus block could significantly reduce the nausea and vomiting, while suprascapular nerve anesthesia and intra-articular local anesthesia can’t reduce the nausea and vomiting compared with the non-pain controlled group [43]. Hadzic et al. have identified that NBA can reduce the PONV compared with the general anesthesia for patients undergoing outpatients rotator cuff surgery [44]. During podiatric surgery in children, patients managed with EA present a higher risks for adverse events, including PONV and urine retention [18]. However, a previous meta-analysis has summarized that patients managed with NBA present a lower incidence of urine retention than patients undergoing EA, but there is no difference in the incidence of PONV [45]. With an updated meta-analysis, NBA was identified to put a significant lower effects on the occurrence rates of PONV, urine retention, and sore throat compared with patients managed with SA, EA, GA, and GA + ISB during the perioperative period of orthopedic surgery in the current study. All of these findings indicated that NBA might perform a better outcome on the prognosis of patients undergoing orthopedic surgery.

ISB is one of the most reliable and commonly anesthetic method applied for the upper extremity with less opioid consumption and opioid-associated adverse effect [46]. Meanwhile, LIA is a safety and effective method for pain control during the perioperative periods of knee and hip surgery [47]. In this study, patients managed with ISB and LIA presented a significant lower occurrence rate of back pain compared with patients undergoing EA, indicating that ISB and LIA might play a better outcome for relieving back pain during orthopedic surgery. Adersen et al. have revealed that LIA presents a superior outcome with less adverse effect, including pain control, than EA during total knee arthroplasty [48]. Another study has also demonstrated that LIA performs a better outcome in pain controlling during total knee arthroplasty [49]. These findings demonstrated LIA and ISB might perform effective roles in relieving pains, such as back pain, during the perioperative period of orthopedic surgery. Despite of these, LIA was also identified to play critical role in relieving headache during the perioperative period of orthopedic surgery, but no statistically difference was identified compared with other group. Therefore, further investigation with large sample size might be required.

Although this study was the first to compare the effects of different anesthesia methods on the complications of orthopedic surgery, but there were still some limitations in this study. First, due to the incomplete data in studies, correction of concomitant variables was not performed, which might affect the results identified in this study. Meanwhile, the subgroup analysis was also not conducted. Second, limited by the property of ADDIS, the calculation of pooled effect size might be influenced. Third, some complications, such as headache and back pain, were not reported in several anesthesia methods; thus, there might be some bias contained in this study.

## Conclusions

In conclusion, according to the network analysis, NBA was a superior anesthesia method in reliving the occurrence of PONV, urine retention, and sore throat compared with patients managed with SA, EA, GA, and GA + ISB during the perioperative period of orthopedic surgery. ISB and LIA were two effective anesthesia methods in lowering the occurrence rate of back pain during the perioperative period of orthopedic surgery. Therefore, it is important to surgeons to select appropriate anesthesia methods during the perioperative period of orthopedic surgery according to the physical fitness of patients and the effects of anesthesia methods on the occurrence of complications.

## Abbreviations

CI: confidence interval
EA: epidural anesthesia
ISB: interscalene block
LIA: local infiltration analgesia
NBA: nerve block analgesia
OR: odd ratio
PSRF: potential scale reduction factor
SA: spinal anesthesia
